# Derivation of Neural Stem Cells from Human Adult Peripheral CD34+ Cells for an Autologous Model of Neuroinflammation

**DOI:** 10.1371/journal.pone.0081720

**Published:** 2013-11-26

**Authors:** Tongguang Wang, Elliot Choi, Maria Chiara G. Monaco, Emilie Campanac, Marie Medynets, Thao Do, Prashant Rao, Kory R. Johnson, Abdel G. Elkahloun, Gloria Von Geldern, Tory Johnson, Sriram Subramaniam, Dax Hoffman, Eugene Major, Avindra Nath

**Affiliations:** 1 Translational Neuroscience Center, National Institute of Neurological Disorders and Stroke, National Institutes of Health, Bethesda, Maryland, United States of America; 2 Section of Infections of the Nervous System, National Institute of Neurological Disorders and Stroke, National Institutes of Health, Bethesda, Maryland, United States of America; 3 Laboratory of Molecular Medicine and Neuroscience, National Institute of Neurological Disorders and Stroke, National Institutes of Health, Bethesda, Maryland, United States of America; 4 Molecular Neurophysiology and Biophysics Unit, Eunice Kennedy Shriver National Institute of Child Health and Human Development, National Institutes of Health, Bethesda, Maryland, USA; 5 Laboratory of Cell Biology, Center for Cancer Research, National Cancer Institute, National Institutes of Health, Bethesda, Maryland, United States of America; 6 Bioinformatics Section, National Institute of Neurological Disorders and Stroke, National Institutes of Health, Bethesda, Maryland, United States of America; 7 Cancer Genetics Branch, National Human Genome Research Institute, Bethesda, Maryland, United States of America; Temple University School of Medicine, United States of America

## Abstract

Proinflammatory factors from activated T cells inhibit neurogenesis in adult animal brain and cultured human fetal neural stem cells (NSC). However, the role of inhibition of neurogenesis in human neuroinflammatory diseases is still uncertain because of the difficulty in obtaining adult NSC from patients. Recent developments in cell reprogramming suggest that NSC may be derived directly from adult fibroblasts. We generated NSC from adult human peripheral CD34+ cells by transfecting the cells with Sendai virus constructs containing Sox2, Oct3/4, c-Myc and Klf4. The derived NSC could be differentiated to glial cells and action potential firing neurons. Co-culturing NSC with activated autologous T cells or treatment with recombinant granzyme B caused inhibition of neurogenesis as indicated by decreased NSC proliferation and neuronal differentiation. Thus, we have established a unique autologous *in vitro* model to study the pathophysiology of neuroinflammatory diseases that has potential for usage in personalized medicine.

## Introduction

T cell activation plays an important role in inflammation-related neuronal injury associated with diseases including encephalitis, the progressive forms of multiple sclerosis [1–3] and a wide variety of other neuroinflammatory diseases. Once infiltrated in the brain, inflammatory factors released from T cells may injure neurons or impair the normal functions of local neural stem cells, resulting in loss of functional neurons and delay of recovery [4,5]. We have previously reported that granzyme B (GrB) released from activated T cells inhibits neurogenesis in adult animals and in cultured human fetal neural stem cells. This suggests that GrB-inhibited neurogenesis may play an important role in the pathophysiology of T cell-related neurological disorders [6]. However, the role of such mechanisms in disease pathogenesis is still uncertain due to lack of access to adult neural stem cells and autologous T cells. Furthermore, the genetic background of an individual may dictate the degree to which activated T cells may impair neurogenesis. Thus, it is important to obtain neural stem cells from individual patients to address these issues.

While obtaining neural stem cells from human adult brain is not routinely feasible, recent developments in regenerative medicine, especially the generation of induced pluripotent stem cells (iPSC) from somatic cells, provide novel opportunities to generate neural cells from these stem cells. Human adult multipotent stem cells can be generated from diverse tissues such as skin, bone marrow and adipose tissue [7–10]. However, in most cases, the number of the adult stem cells obtained is very limited and requires extended periods of time for expansion of cells, thereby limiting their usefulness within the context of personalized medicine. Following the initial report of generation of iPSCs from mouse and human fibroblasts using four transcription factors (Sox2, Oct3/4, Klf4, and c-Myc) [11,12], iPSCs have been generated from fibroblasts of patients with neurological diseases which were then differentiated into neurons successfully [13–15]. Still, the processes to differentiate neurons from ES/iPSC usually involve embryoid body formation [16] or more recently by inhibiting SMAD signals using small molecules [17]. These processes involving iPSC generation are time and labor consuming, and may not represent physiological neurogenesis. Several recent reports indicate that neural stem/progenitor cells can be directly generated from skin fibroblasts [18–20]. The ability to generate neural stem cells directly without the need to generate iPSCs is a major advancement in studying neurogenesis in diseased states because the neural stem cells are self renewing and can be expanded and differentiated into neurons and glia. The direct conversion would result in substantial time and cost savings. Hence we investigated the generation of neural stem cells from CD34+ hematopoietic stem cells, which represent more convenient alternatives to fibroblasts. 

In this study, we used Sendai virus constructs encoding four iPSC transcriptional factors (Sox2, Oct4, Klf4 and c-Myc) to derive monolayer adherent neural stem cells from CD34+ cells from both cord blood cells and adult peripheral blood. The generated neural stem cells could be further differentiated to functional neurons and glial cells and were used successfully as a model to study inflammation-related neurogenesis. 

## Results

### Generation of neural stem cells from cord blood CD34+ cells

**Figure 1:**
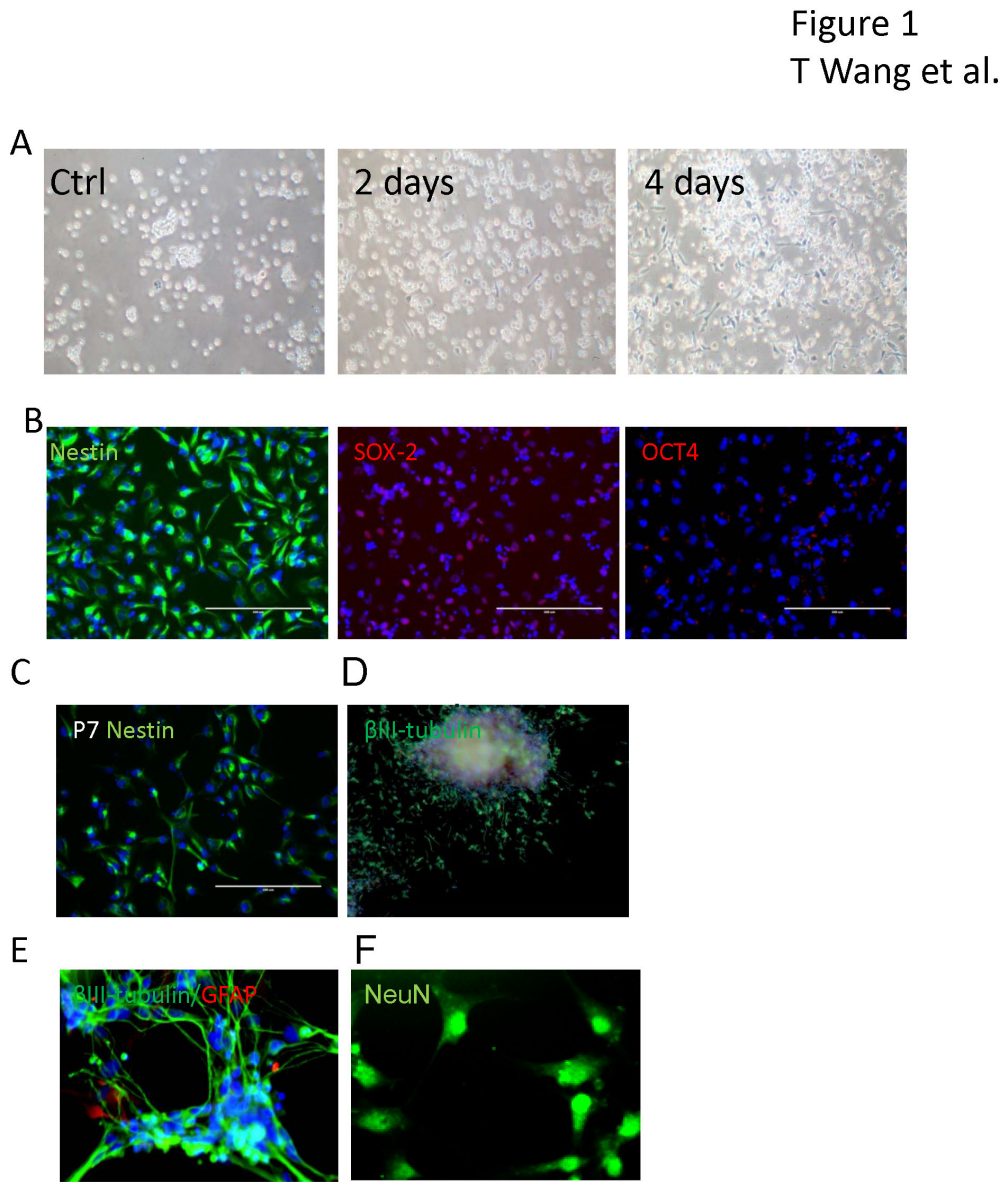
Generation of neural stem cells from cord blood CD34+ cells. (A) Non adherent cord blood CD34+ cells (Ctrl) were transduced with Sendai virus constructs containing Oct3/4, Sox2, Klf4 and c-Myc and maintained in neural stem cell medium. Adherent cells were observed on day 2 which proliferated rapidly through day 4 post-infection. (B) More than 95% of the generated cells immunostained for nestin (green) and SOX-2 (red), but were OCT4 negative. Cell nuclei were counterstained with DAPI (blue). The cells were subcultured up to passage 7, when a decline in nestin positive cells was first noticed (C). Spontaneously differentiated neuronal like cells were observed when incubated in neural stem cell medium for over a week without changing medium. These cells developed neural sphere like structures from which βIII-tubulin positive cells with long processes grew out (D). When neural stem cells derived from CD34+ cells were placed in neural differentiation medium for two weeks, both βIII-tubulin (green) neurons and GFAP (red) positive astroglia were seen, along with some cell aggregates (E). Immunostaining for NeuN (green) in the nuclei confirms the presence of relatively mature neurons (F).

CD34+ cells derived from cord blood were cultured in StemSpan Serum-Free Expansion Medium (SFEM) and expanded for four days. The cells remained non-adherent without any significant aggregation (Figure 1A). To determine whether Sendai viral vectors encoding four iPSC transcriptional factors (Sox2, Oct3/4, Klf4 and c-Myc) could generate neural stem cells from cord blood CD34+ cells, the cells were infected with the virus at a multiplicity of infection (MOI) of 3 after five days in culture. As seen in Figure 1A, two days after infection, adherent cells with bipolar morphology were observed and clone-like aggregates appeared. The cells expanded quickly and the adherent cells reached 30-40% confluence after 4 days of infection while in non-transfected controls no adherent cells were observed. To expand the neural stem cells, we collected the adherent cells by gentle pipetting and transferred the cells to poly-D-lysine coated plates in neural progenitor cell medium. The cells were passaged when they reached 60% confluence. To characterize these cells, immunostaining for nestin, OCT4 and SOX-2 were performed at passage 3 (Figure 1B). More than 95% of the cells were nestin positive and SOX-2 positive. No nuclear OCT4 staining was observed. This indicated that the cells were not iPSCs but expressed markers of neural stem cells. Following serial passaging of these cells we found that there was a decrease in level of nestin staining in cells at passage 7 (Figure 1C). For further experiments, we used cells at passage 3. We observed that some neural stem cells spontaneously differentiated into βIII-tubulin positive cells when incubated in neural stem cell medium for over a week. These cells developed neural sphere like structures and βIII-tubulin positive cells with long processes outgrowing from the cell aggregates but no glial fibrillary acidic protein (GFAP) positive cells were observed, indicating that the neural stem cells generated from cord blood CD34+ cells have the potential to differentiate to neurons spontaneously (Figure 1D). To achieve more homogenous and controllable neural differentiation, we collected the neural stem cells by mechanical dissociation and plated the cells in neuronal differentiation medium. After two weeks of differentiation, we detected βIII tubulin positive neurons and a few GFAP positive astroglial cells (Figure 1E). The morphologies of these neurons were more complex with multiple long processes when compared with spontaneously differentiated neurons (Figure 1D). These neurons were also NeuN positive (Figure 1F), indicating that the process of induction generated relatively mature neurons and glial cells from the neural stem cells. 

### Derivation of neural stem cells from adult CD34+ cells from peripheral blood

**Figure 2:**
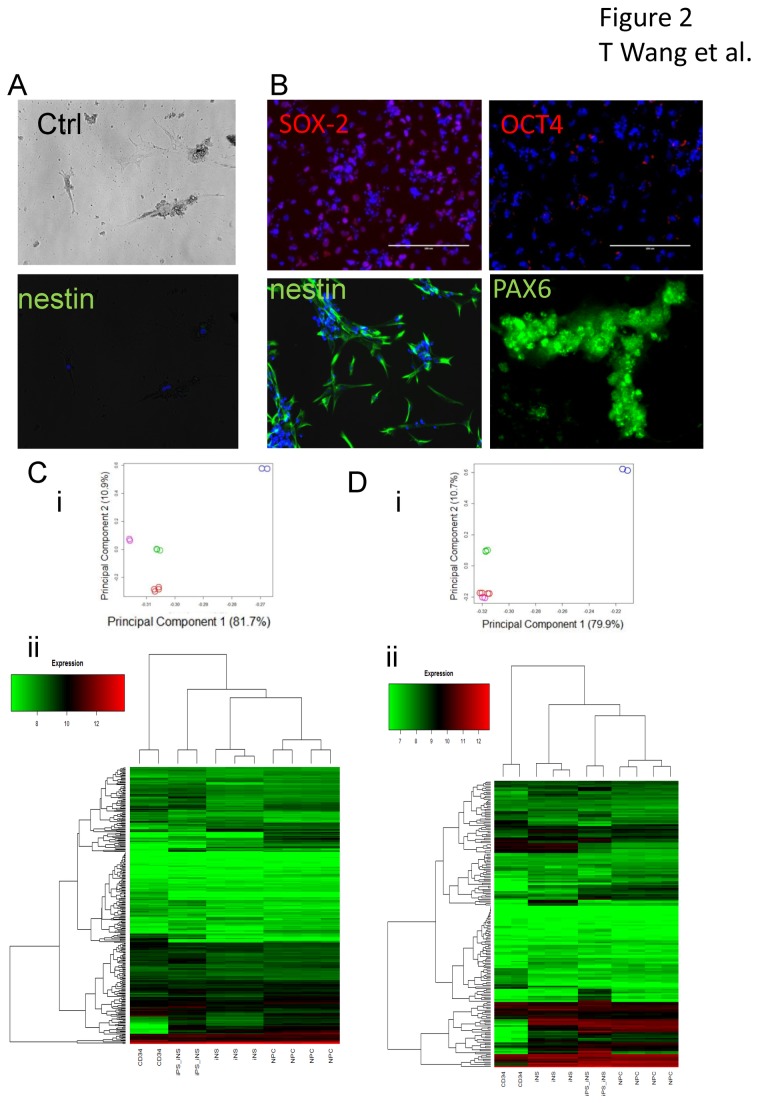
Generation of neural stem cells from adult peripheral CD34+ cells. Adult peripheral CD34+ cells were transduced using Sendai virus constructs containing Oct3/4, Sox2, Klf4 and c-Myc. (A) A few adherent cells were observed in uninfected CD34+ cell cultures (Ctrl) in neural stem cell medium. These cells did not show any proliferation and or nestin immunostaining. (B) Following transduction and culture in neural stem cell medium, CD34+ cells resulted in more adherent proliferating cells, which were SOX-2 (red), nestin (green) and PAX6 (green) positive, but negative for OCT4 (red). Cells were counterstained with DAPI (blue). Gene expression profiling were studied using samples from one CD34+ cells culture (two technical repeats), one iPS differentiated neural stem cells culture (iPS-iNS, two technical repeats), two induced neural stem cells cultures (iNS, one with two technical repeats) and three human primary fetal neural progenitor cells cultures (NPC, one with two technical repeats). Sample-to-sample relationships based on covariance-based Principal Component Analysis (i) and correlation-based clustering analysis (ii) using 311 markers for pluripotency (C) and 197 neuronal progenitor markers (D) (NCBI PMID: 23117585).  Both analyses were performed in R (http://cran.r-project.org/) using the princomp and heatmap.2 functions respectively.  Samples depicted in the PCA scatterplot are represented as circles and described by type using color (CD34 = blue, iPSC-iNS = pink, iNS = green, NPC = red).  Marker expression is of type RMA (log, base=2).

To determine whether neural stem cells could be generated from CD34+ cells from adult peripheral blood cells, we used two methods to purify CD34+ cells from the peripheral blood: negative selection, which resulted in purer hematopoietic progenitor cells by depleting some non-hematopoietic CD34+ cells such as B lymphocyte precursors and a sub-population of dendritic cells, and positive selection using anti-CD34 antibody Dynabeads. We did not observe drastic differences in the efficiency of neural stem cell generation between the two methods, partly due to the limit of repeats and considerable variability in the efficiency between different samples. We increased the MOI from 3 to 20 when using Sendai virus for gene transduction. After transduction, we noticed a slightly slower cell proliferation compared to CD34+ cells from cord blood cells. We also observed a few adherent cells in control cultures without virus transfection. These cells had irregular shapes and did not proliferate compared to the adherent cells induced by virus transfection. To determine the identity of these cells, we treated the cells with neural progenitor cell medium in the same wells without detaching and replating the cells for 1 week. As seen in Figure 2A, the few adherent cells in control wells were nestin negative, while the adherent cells in transduced wells were mostly SOX-2, nestin and PAX6 positive (Figure 2B). Thus, transduction was necessary for successful neural stem cell derivation. We later used multiple replating of CD34+ cells to avoid contamination with any attached cells. We further compared the gene expression profiles of induced -neural stem cells (iNS) with adult CD34+ cells, primary cultured human fetal neural progenitor cells (NPC) and neural stem cells differentiated from an iPSC cell line (iPS-iNS) derived from adult CD34+ cells (pluripotent and neural progenitor genes were listed in Table S1). Almost identical gene expression profiles were observed within iNS lines, indicating the iNS generation process generated cells with a consistent identity (Figure 2C and D). With regards to pluripotent gene expression, there were significant differences between CD34+ cells and iNS cells, the latter were closer to NPCs than iPS- iNS (Figure 2C), In contrast, the expression pattern of neural progenitor cell specific genes were similar among iNS cells, iPS-iNS cells and NPCs (Figure 2D). There are 67 genes out of 196 that showed an absolute difference in means >= 1.5X between iNS and NPCs, but most of the differences were within two folds (Figure S1). Furthermore, PAX6 expression was also confirmed in both iPS-iNS and iNS using Western-blot assay (Figure S2). To further characterize the genes differentially expressed between iNS and the original CD34+ cells, we run the Gene Set Enrichment Analysis [21] (GSEA) to compare iNS with gene signatures generated from previously published neural stem cells. The gene signature of direct generated neural stem cells from human fibroblasts and the gene signature of neural stem cells from subventricular zone of 3rd ventricle[22] both show high enrichment score (ES) and statistical significance (Figure S3),. 

### Derivation of functional neurons from adult CD34+ cells generated neural stem cells

**Figure 3:**
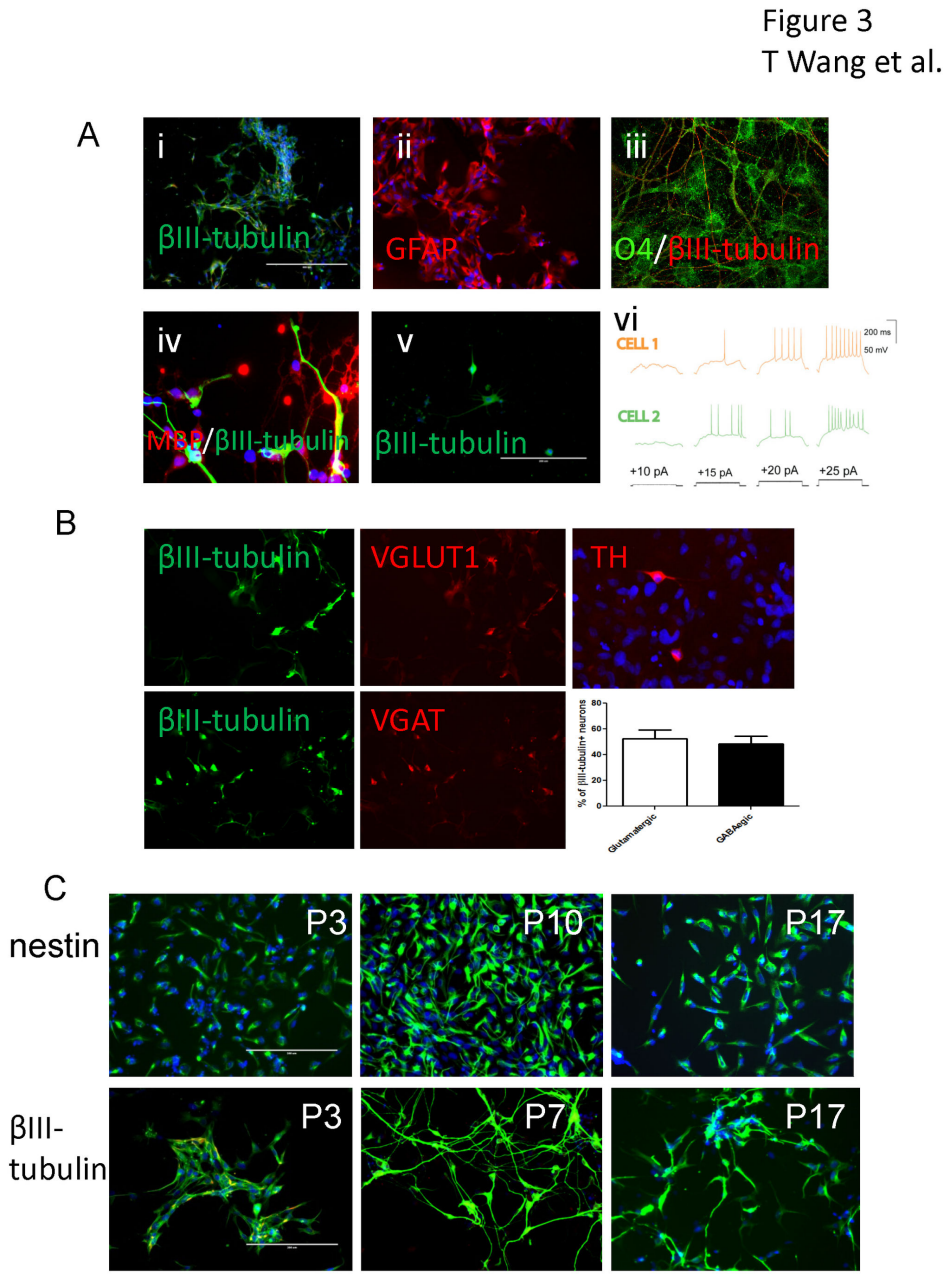
Neurons with action potentials and glial cells differentiated from induced neural stem cells from adult peripheral CD34+ cells. The induced neural stem cells differentiated into βIII- tubulin positive neurons (green, Ai) in neuronal differentiation or GFAP positive astroglia (red, Aii) in astroglial differentiation medium for 1 week. Oligodendrocyte progenitor differentiation was achieved by incubating the neural stem cells with oligodendrocyte differentiation medium. O4 positive cells (green, Aiii, after 2 weeks) and a few of MBP positive cells (red, Aiv, after four weeks) were detected by immunostaining. Spontaneous neuronal differentiation was also observed in low concentration seeded neural stem cell culture (1000 cells/well in a 24 well plate) after incubation in neural stem cell medium for 1 week (Av). Action potentials were recorded from neurons differentiated for 2 weeks using whole cell patch clamp. Action potentials recorded on two separated cells are shown (Avi). Double staining with either VGLUT1 or VGAT with βIII- tubulin showed most βIII- tubulin positive neurons were either glutamatergic or gabaergic neurons while a few of doparminergic neurons were also detected by TH immunostaining after incubation in neuronal differentiation medium for 2 weeks (B). The induced neural stem cells were passaged for 17 passages and immunostained for nestin as a neural stem cell marker. The cells were also cultured in neural differentiation medium for 2 weeks and immunostained for βIII-tubulin to determine their neuronal differentiation capabilities (C).

Our protocol for generation of neurons involves the transduction of CD34+ cells with Sendai virus, conversion to neural stem cells aided by incubation in neural stem cell medium, where cell populations can be expanded, and then incubation in neuronal differentiation medium. Astroglia and oligodendrocyte progenitor differentiation were induced by incubation in corresponding differentiation media. βIII-tubulin positive neurons and GFAP positive astroglia were observed after one week of induced differentiation (Figure 3Ai and ii). Oligodendrocyte progenitor marker O4 positive cells were observed after two weeks of differentiation (Figure 3Aiii) and a few myelin basic protein (MBP) positive cells were observed after 4 weeks of differentiation (Figure 3Aiv). MBP production was also confirmed using Western-blot assay and (Figure S2). However, when neural stem cell were seeded at 1000 cells/ well in a 24 well plate for 1 week in neural stem cell medium, a few βIII- tubulin positive cells with typical neuronal appearance were also observed (Figure 3Av). To determine whether differentiated neurons from the adult CD34+ cell-induced neural stem cells were functional, we used whole-cell patch clamp to record action potentials from these cells. As seen in Figure 3Avi, after two weeks of differentiation, action potentials could be recorded in the cells in response to current injection. We further determined the neuronal cell types by immunostaining βIII-tubulin positive cells with antibodies to vesicular glutamate transporter 1 (VGLUT1), vesicular GABA transporter (VGAT) or tyrosine hydroxylase (TH). We found most neurons were glutamatergic neurons or gabaergic neurons (each representing about 50% of βIII-tubulin positive cells, Figure 3B). Some TH-positive doparminergic neurons were also induced in the neural stem cell culture by treatment with neural differentiation medium for 2 weeks (Figure 3B). We noticed that passaging of the cells on poly-D-lysine coated surface inhibited cell proliferation and caused excessive cell differentiation, thus we used non-coated plates for maintaining the iNS cultures. We found that passaging and maintaining cells at low level of confluence (< 60%) combined with no coating of the surface of the culture dishes was necessary to keep the contaminated/differentiated cells at low level. This way, the cells have been cultured for 17 passages without losing nestin staining and neuronal differentiation capabilities (Figure 3C). Beyond that, as shown in Supplementary Materials (Figure S4), most of the cells were still positive for neural stem cell surface markers CD15 or CD24 at passage 42, with few cells were CD44 positive, a marker for astroglial differentiation, indicating these cell markers could be used for neural stem cell sorting by using flow cytometry if necessary.

### Confirmation of stemness and tri-potency of induced neural stem cells

We further confirmed the stemness and tri-potency of the induced neural stem cells using collagen semisolid culture. The semisolid condition prevents cell aggregation and thus favors colony formation from single cells. We found that about 1% of the cells formed primary neurospheres while 1.5% of the single cells dissociated from primary neurospheres formed secondary colonies. Furthermore, dissociated cells from a single secondary neurosphere were differentiated into βIII-tubulin and GFAP positive cells in astroglial differentiation medium and O4 positive cells in oligodendrocyte differentiation medium ( Figure S5). These observations confirmed the stemness of the induced neural stem cells and their potential to generate all three lineages of neural cells. 

### GrB inhibits neurogenesis in induced neural stem cells

**Figure 4:**
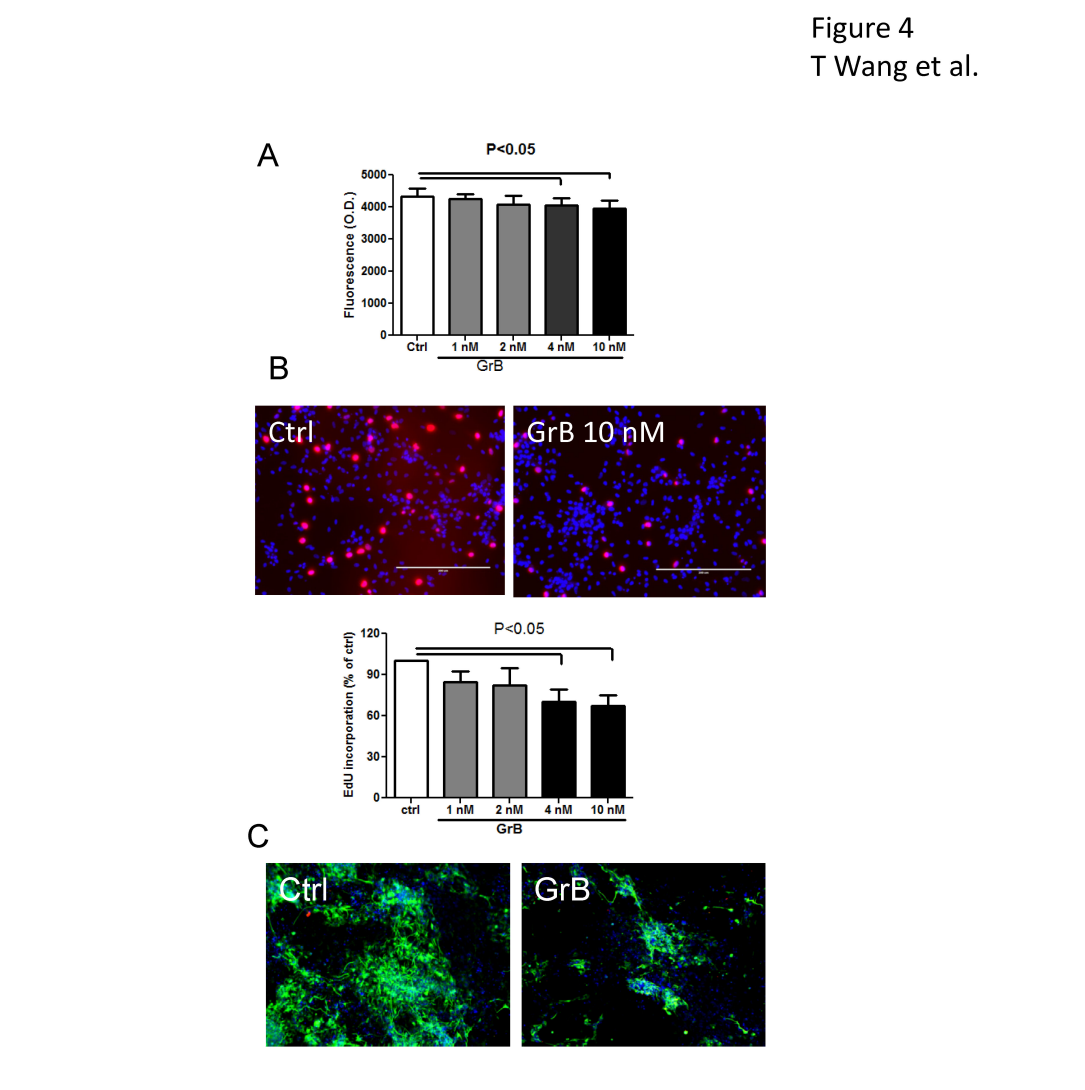
Inhibition of neurogenesis of induced neural stem cells by inflammatory factor, GrB. Following treatment with GrB, (A) the total number of induced neural stem cells was determined by using cellquanti-blue assay and (B) the number of proliferating cells was determined using an EdU incorporation assay. Representative photomicrographs show EdU (red) incorporation and DAPI stained nuclei (blue). Results are presented as mean±SEM from at least three independent experiments (n=4 for A and n=3 for B). (C), the effect of GrB on neuronal differentiation was studied using induced neural stem cells derived from adult peripheral CD34+ cells. GrB was used to treat the cells in neuronal differentiation medium for 1 week and βIII-tubulin immunostaining (green) was used to characterize the neurons. GrB treatment resulted in decreased βIII-tubulin positive cells. The morphology of the differentiated neurons in GrB treated group shows that they were more immature, with shorter neurites and less complicated networks, compared to untreated control.

We previously reported that activated T cells inhibit neurogenesis in primary cultured human fetal neural stem cells via the extracellular release of GrB [6]. To determine whether induced neural stem cells behave the same way as the fetal neural stem cells in inflammatory condition, we treated the induced neural stem cells from cord blood with recombinant GrB in 96 well plates for 24 hours. We used cellquanti blue assay to determine the cell numbers. As shown in Figure 4A, a minor but statistically significant decrease in the cells number was observed in treatments with GrB 4 nM (4045±184) and 10 nM (3959±182) compared to control (4340±180). To determine whether the effect was due to the inhibition of cell proliferation, we treated the cells with GrB for 24 hours and during the last 4 hours 5-ethynyl-2’-deoxyuridine (EdU) reagent was added which is incorporated into dividing cells. By determining the ratio of EdU positive cells among the 4',6-diamidino-2-phenylindole (DAPI) positive cells in the same field, we found that GrB treatment significantly decreased EdU incorporation in the induced neural stem cells (with 4 nM of GrB at 70.3±9.3% and 10 nM of GrB at 67±8.3% of control), indicating GrB inhibited neural stem cell proliferation (Figure 4B). The effect of GrB on neuronal differentiation was studied by treating neural stem cells with GrB in neural differentiation medium for 1 week. The cells were then immunostained for βIII-tubulin. Statistics were not provided because the actual number of neurons was difficult to count due to the intertwined and complicated networks of neurites in the experimental groups. However, the difference between control and GrB treated groups was apparent as shown in the representative photos. As seen in Figure 4C, GrB treatment resulted in fewer βIII-tubulin positive cells and shorter neurites, indicating that GrB treatment inhibited neurogenesis. 

### Activated autologous T cells inhibit neurogenesis

**Figure 5:**
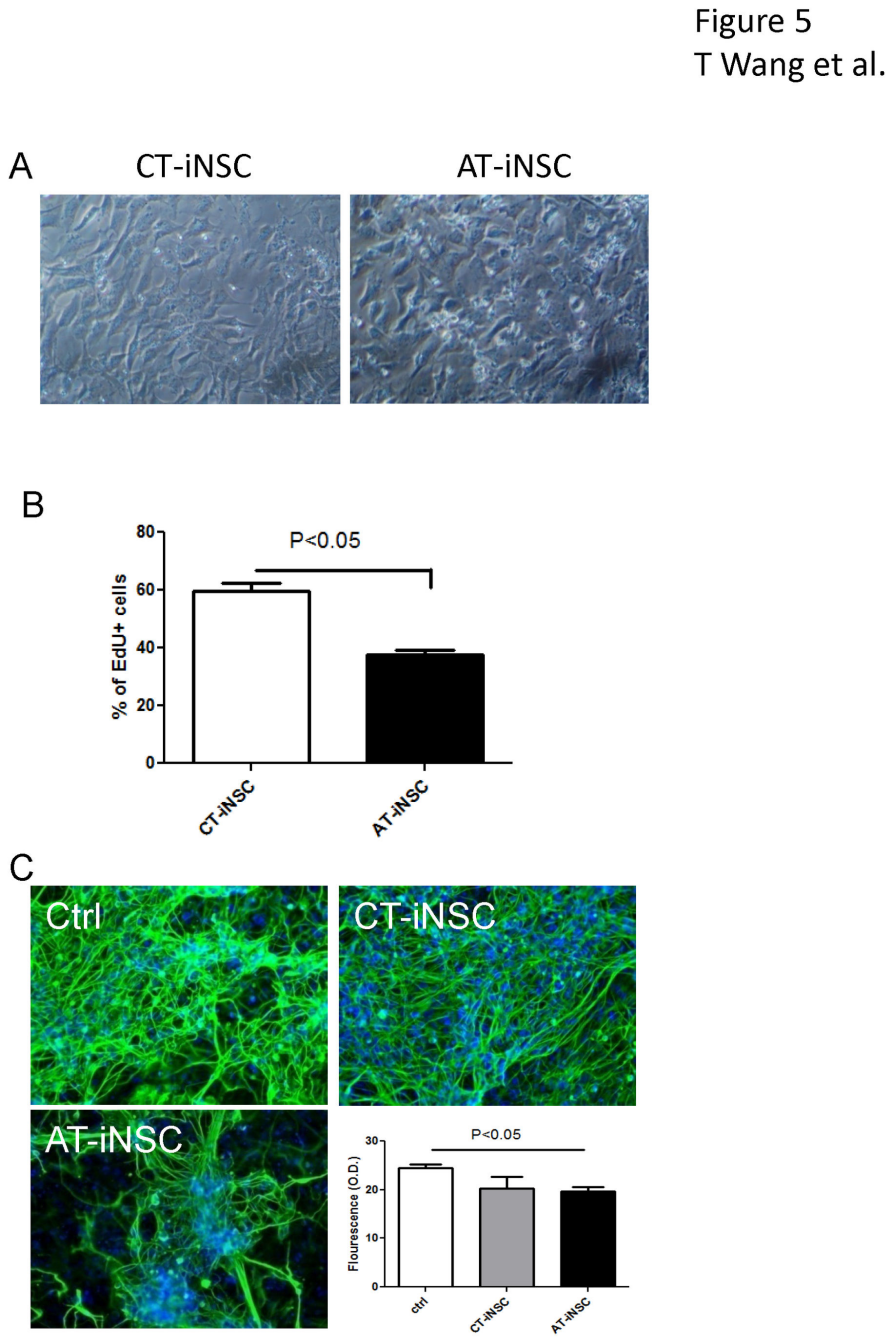
Inhibition of neurogenesis by activated autologous T cells on induced neural stem cells. Induced neural stem cells derived from adult peripheral CD34+ cells were cocultured with restive (CT) or activated autologous T cells (AT) for 24 hours. (A) After washing with PBS three times, no adherent T cells were observed in induced neural stem cells co-cultivated with restive T cells (CT-iNSC), while adherent T cells were observed in cultures of induced neural stem cells with activated T cells (AT-iNSC). (B) EdU incorporation assay was used to determine the proliferation of iNSC after 24 hours of co-culture. (C) After 7 days in neuronal differentiation medium, neuronal differentiation was studied by immunostaining for βIII-tubulin. The fluorescence was detected using a plate reader at excitation wavelength 495 nm and emission wavelength 519 nm. AT-iNSC coculture resulted in significantly decreased neuronal differentiation compared to non co-cultured control. Morphologically, AT treatment resulted in fewer neurons, which were more locally aggregated, compared to CT treated groups.

To study the effect of autologous T cells on neurogenesis, T cells were purified following leukapheresis of healthy volunteers. Restive and CD3/CD28 activated T cells were co-cultured with the induced neural stem cells generated from CD34+ cells from the same leukapheresis. After 24 hours of co-culture, the cultures were washed with phosphate buffered saline (PBS) to remove non-adherent T cells. As shown in Figure 5A, after washing, no adherent T cells were observed in the co-cultures with control restive T cells, while significant amounts of adherent T cells were observed on induced neural stem cells co-cultured with activated T cells. When determining the EdU incorporation, activated T cells (37.3±1.8%) inhibited neural stem cell proliferation significantly compared to control T cells (59.6±3.1%) (Figure 5B). The effect of autologous T cells on neural differentiation was also studied by co-culturing the T cells and induced neural stem cells in neural differentiation medium for 7 days. The neurons were immunostained for βIII-tubulin and the density of βIII-tubulin fluorescence was detected using a fluorescence plate reader. As shown in Figure 5C, while both co-cultivation with resting T cells and activated T cells caused decrease of neuronal differentiation, co-cultivation with activated T cells resulted in more significant morphological changes, including more locally concentrated neuronal differentiation and less complicated neurites networks. 

### Activated T cells interact with neural stem cells

**Figure 6:**
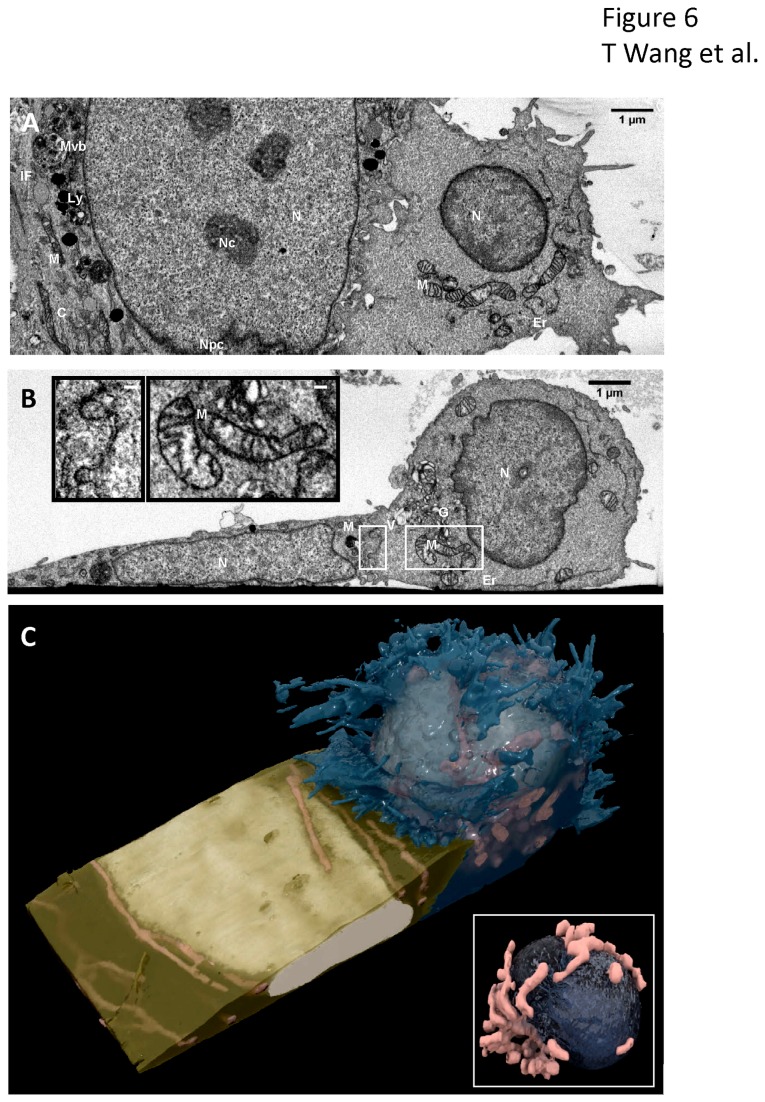
3D imaging of contact between neural stem cell (left) and activated T cell (right) by ion abrasion scanning electron microscopy (IA-SEM). (A, B) Representative, orthogonal 2D images from a 17.57 um x 6.30 um x 8.72 um stack volume show the two cells in direct contact with interlocking cellular membranes in the XZ plane (A) and XY plane (B). The XY plane reveals a localization of Golgi apparatus, vesicles, and mitochondria in the activated T cell near the site of contact. Subcellular organelles are well preserved including nucleus (N), nucleolus (Nc), nuclear pore complexes (Npc), multivesicular bodies (Mvb), lysosome (Ly), mitochondria (M), cytoskeleton (C), intermediate filament (IF), Golgi apparatus (G), vesicles (V), and endoplasmic recticulum (ER). (B, Inset) Expanded view of boxed regions in panel (B) of the interlocking membranes at the cell-cell junction (left) and the T cell mitochondria (right) to illustrate level of detail in images. Scale bars are 100 nm. (C) 3D visualization of the SEM image stack show the activated T cell membrane (blue) engulfing an edge of the neural stem cell membrane (gold). The activated T cell spherical shaped nucleus (ivory), compact mitochondria clusters (pink), and complex membrane protrusions are seen in stark contrast with the neural stem cell flat nucleus (ivory), elongated mitochondria (pink), and smooth cell membrane. (C, Inset) Expanded view of the interior of the T-cell showing the T cell nucleus (navy) is pinched in the center, with the cleft line oriented directly toward the site of contact. Mitochondria (pink) are found clustered along the cleft line.

The ultrastructure of the neural stem cells and activated T cells with cell-cell junctions were observed at nanometer resolution using ion abrasion scanning electron microscopy (also referred to in the literature as focused ion beam scanning electron microscopy or FIBSEM). This revealed that the two cells were in direct contact with each other (Figure 6). 2D electron micrographs (Figure 6A and 6B) showed membrane protrusions from both cells interlocked at the contact site. The Golgi apparatus, vesicles, and mitochondria of the T-cell localized at the cell-cell interface. 3D visualizations (Figure 6C) revealed a cleft line running along the center of the T cell nucleus, surrounded by two compact clusters of mitochondria on polar opposite sides. The cleft line was directly facing the contact region. The T cell membrane engulfed an edge of the neural stem cell at the contact zone. These evidences suggest that the contact include active processes that likely involve participation of cytoskeletal elements. The stark structural difference between the neural stem cell flat nucleus, elongated mitochondria, and smooth cell membrane and the activated T cell spherical shaped nucleus with central cleft line, compact mitochondria clusters, and complex membrane protrusions provide morphological clues to the activation states of the two cells. These observations suggest that neural stem cell derived from CD34+ cells co-cultured with activated autologous T cells result in active and functionally relevant cell-cell interactions. 

## Discussion

Understanding the factors that modulate neurogenesis in pathological conditions is critical to study disease pathophysiology and to develop treatments for most neurological disorders. However, the lack of neural stem cells from patients remains a major road block for such studies. We used Sendai virus constructs containing Yamanaka’s four transcriptional factors to generate adherent neural stem cells from CD34+ cells from both cord blood and adult peripheral blood and showed that these cells responded to inflammatory assault in a manner similar to human fetal neural stem cells. 

 Adult brain neurogenesis plays an important role in memory formation, normal learning activity and maintenance of the brain volume [23–25]. Further, dysfunction of neural stem cells may play a role in the pathophysiology of a variety of neurological diseases such as multiple sclerosis [26], HIV-1 associated cognitive disorders [27], autism [28] and depression [29]. Several studies show that inflammatory responses can modulate neural stem cell functions in the brain. Stem cells generally inhibit immune or inflammatory reactions [30–32], on the other hand, a pro-inflammatory environment may impair neural stem cell functions, leading to inhibition of neuronal differentiation [6,33,34]. The residential inflammation/immune responses may also cause failure of stem cell transplantation as a therapy for the neuroinflammatory disorders [34]. Compared to the effect observed in animal studies, which usually have a homogeneous genetic background, patient populations have a wide spectrum of genetic backgrounds, which may result in the inconsistency of clinical symptoms and responses. Neuronal cells from individual patients are thus the best models to address these challenges.

Because it is almost impossible to obtain neural stem cells directly from adult brain, alternative approaches are necessary. Neural stem cells can be differentiated from iPSC or stem cells obtained from a variety of tissues from the human body, such as exfoliated deciduous teeth, adult skeletal muscles [35,36], bone marrow [37] and skin [38] amongst others. These stem cells could then be used to generate neural progenitor cells or immature neuronal cells. However, it is still questionable whether neural stem cells derived from sources other than neural tissues can generate functional neurons. For example, neuronal makers and neuronal like cells were generated from human bone marrow stromal cells and CD133+ stem-like cells but failed to generate fast sodium currents or functional neurotransmitter receptors [39]. Compared with obtaining stem cells or generating iPSC, induced neural stem cells from easily accessible cells are more desirable for clinical personalized medicine. It has been reported that neural sphere-like cells or neural stem/progenitor cells could be directly generated from human fibroblasts [18,40] using stem cell transcriptional factors. Hence, we explored the possibility of generating neural stem cells from hematopoietic stem cells without the intermediary step of generating iPSC. CD34+ hematopoietic stem cells also express some neural genes [41] and neural stem cells also exhibit hematopoietic potential [42]. When cultured with astroglial conditional media for 20 days, bone marrow derived adult CD34+ cells differentiated into cells with neuronal phenotypes, including cells with nestin expression [43]. Although the neuronal phenotypes and functions in the differentiated cells are still questionable [44–46], this evidence indicates that CD34+ cells may be easier to convert to functional neural stem cells compared to fibroblasts. Furthermore, as peripheral blood mononuclear cells or lymphocytes can be easily collected and purified at the same time as CD34+ cells, the immune cells could be co-cultured with generated neural stem cells or neurons without concern about major histocompatibility complex mismatch since both cell types would have the same genetic background. This provides a perfect tool to study the effect of immune reactions on neurogenesis and neurodegeneration. 

Compared to recombinant retrovirus and lentivirus, recombinant Sendai virus is more efficient in transferring genes to CD34+ cells without DNA integration [47]. The method has been used successfully to generate iPSC from adult CD34+ hematopoietic progenitor cells [48]. Thus we chose the Sendai virus system to transduce CD34+ cells and generate neural stem cells. We first transfected cord blood CD34+ cells and noticed that adherent cells appeared after 2 days of the transduction . These cells were mostly bipolar cells and did not maintain the phenotype of stem cells nor did they expand clonally. After 5 days, the adherent cells were collected and expanded in neural stem cell medium. Given the short period after transduction, the non-iPSC cell culture conditions, and the apparent morphological differences, it is very likely that the cells generated were derived without the stable iPSC stage. This was further confirmed by immunostaining, which showed that the cells were positive for the neural stem cell markers, nestin and SOX-2 but not OCT 4, an important stem cell maker, the expression of which, inhibits neuronal differentiation but induces pluripotency in neural stem cells [49–51]. As Oct3/4 was one of the genes used for transduction, it may be transiently expressed in these cells and got lost quickly in the neural stem cell culture conditions, similar to that seen with the generation of neural stem cells from fibroblasts [19]. Oct3/4 was used although the neural stem cells did not express this gene. Oct3/4 activation is known to regulate the expressions of other transcription factors involved in stem cell maintaining and pluripotency [52]. Based on previous report which transformed fibroblasts to neural stem cells, the efficiency of neural stem cell generation was significantly lower without intrinsic/induced Oct3/4 expression during the first 5 days of induction, indicating Oct3/4 is helpful in increasing the efficiency of neural stem cell generation at an initial stage to provoke transit stem cell gene transcriptions, though a real stable iPSC stage was not necessary (see review [53]). The generated adherent neural stem cells from cord blood CD34+ cells were passaged up to five times without significant loss of nestin immunostaining. The nestin staining in the adherent cells started decreasing at passage 7, which is similar to that seen with our primary cultures of human fetal neural stem cells [6]. When CD34-derived neural stem cells were cultured in neural differentiation medium containing cyclic AMP, brain-derived neurotrophic factor (BDNF) and glial cell-derived neurotrophic factor (GDNF), the cells differentiated to βIII-tubulin positive neurons and GFAP positive astroglia, some of the neurons also expressed NeuN, a marker for mature neurons. 

Generally, converting adult cells is more difficult than embryonic or cord-blood cells. Thus we increased the MOI of the virus when infecting adult CD34+ cells. Similar to the neural stem cells generated from cord-blood CD34+ cells, neural stem cells generated from adult peripheral blood CD34+ cells were nestin, PAX6 and SOX-2 positive but OCT4 negative. We were aware that other mesenchymal stem cells (MSC) if transfected may also give rise to neural stem cells using our method. However, the chance of contamination of other stem cells was low in our experiments. The number of stem cells in peripheral blood is very low. Without purification, it will be very difficult to have enough cells to generate neural stem cells using the viral titers in our protocol. Actually, we have tried using peripheral blood mononuclear cells (PBMC) without CD34+ purification to generate neural stem cells but without success. We used cord-blood CD34+ cells and negative selection purified adult CD34+ cells for our concept approving experiments. The cord blood CD34+ cells were 98% pure and the adult CD34+ cells were up to 97% pure. We excluded the MSC contamination based on immunostaining of CD34+ cells using some of the specific markers to detect MSC. In fact, CD29 and CD271, known to be expressed on MSC cells ([54–57]) were negative on the enriched CD34+ cells (Figure S6). The 2-3% of contamination would most likely consist of differentiated blood cells. Thus we concluded that other mesenchymal stem cells were not responsible for the constant neural stem cell generation in our experiments. The neural stem cells generated from adult CD34+ cells were able to differentiate to βIII-tubulin positive neurons, among them most were glutamatergic or gabaergic neurons. Furthermore, we also detected a few (<1%) tyrosine hydroxylase positive cells, indicating that neural stem cells could differentiate to doparminergic neurons without a specific neuronal type induction. We determined whether we could derive functional neurons from the induced neural stem cells by electrophysiology. Prolonged differentiation for more than 2 weeks produced neurons capable of generating action potentials, indicating that they were functional mature neurons. We compared the gene expression profiles of thus generated neural stem cells with adult CD34+ cells, human NPC and iNS differentiated from iPSC using two pre-determined gene sets, pluripotent genes and neural stem cell genes, according to previous report [58]. We found almost identical expression profiles between the iNS cells we generated, although they were at different passages (P7 and P17) and from CD34+ cells from different donors. This indicates the iNS generation protocol is capable of generating neural stem cells consistently, and the generated cells could maintain their neural stem cell properties during long term culture and passages. When comparing the neural progenitor gene profiles between iNS and NPCs, most of the differences were within two folds. For those with differences bigger than 2 folds, genes from LOC100272216 contributed a lot to the difference (8 out of 34 genes, Figure S1). Although no report has been found on the function of this Non-coding RNA expression, increased LOC100272216 expression has been found in prefrontal lobe using BioGPS searches[59] and its expression is lost during erythroid CD34+ cell differentiation (http://www.ncbi.nlm.nih.gov/gds?LinkName=geoprofiles_gds&from_uid=32486693). It is likely that the different gene expression patterns may result from culture conditions and the different development stages of the cells. Also, difference in original tissues (mixed fetal brain tissue for NPC purification) and culture processes may have also introduced some differences. We do not expect such differences will have a significant effect when using the cells for neural development or neuropathogenesis studies. Interestingly, by using GESA analysis, we found that the iNS generated from CD34+ cells were correlated very well with other reported neural stem cells such as directly generated neural stem cells from human fibroblasts and primary cultured adult mouse neural stem cells from subventricular zone of 3rd ventricle, suggesting that both direct induction methods generated similar neural stem cells. Furthermore, the PAX6 expression, central nervous system neuronal differentiation capability and lack of CD44 (highly expressed on certain neural crest cells and neural crest differentiated cells [60,61]) expression in even passage 44 iNS suggest most of our cells are not neural crest cells but neural stem cells. Although we cannot rule out the possibility that a very limited number of neural crest stem cells could be generated using our neural stem cell medium, which was optimal for neural stem cell culture but not inhibitory for neural crest cells. To permit comparison of gene profiles of future lines, we have deposited the microarray data on our lines (see methods section).

We then tested whether the induced neural stem cells could be used to study the effect of inflammation on neurogenesis. By using recombinant GrB to treat neural stem cells, we found that 24 hours of treatment decreased neural stem cell numbers by inhibiting proliferation. While in neuron differentiation medium, GrB treatment decreased the number of generated neurons, as well as the length of neurites from induced neural stem cells derived from adult CD34+ cells. These observations were in agreement with our previous observation in GrB treated primary cultured human fetal neural progenitor cells [6], indicating that the induced neural stem cells behave in a similar way as primary cultured human neural stem cells, at least in the response to GrB-inhibited neurogenesis.

One obstacle to studying the effect of inflammatory cells on neurogenesis is to obtain MHC matched T cells for studying cell-cell contact-dependent mechanisms. As we can generate induced neural cells from peripheral blood, we also purified autologous T cells and studied whether these cells once activated may alter the functional properties of induced neural stem cells. By co-culturing autologous T cells and induced neural stem cells, we found that activated T cells decreased neural stem cell proliferation and neuronal differentiation. These observations were also in agreement with our previous observation using non-autologous T cells [6]. However, restive T cells also decreased neuronal differentiation compared to control. This may be caused by spontaneous T cell activation in *in vitro* cultures. Furthermore, we observed that activated autologous T cells formed a tight interaction with underlying neural stem cells. The scanning electron microscopy findings suggest that the co-cultures of neural stem cells derived from CD34+ cells with activated autologous T cells result in active, and functionally relevant cell-cell interactions, further supporting our hypothesis that activated T cells cause inhibitory effect on neural stem cells. 

In summary, we developed a method which can induce adherent neural stem cells from human adult peripheral blood CD34+ cells. The induced adherent neural stem cells could be easily differentiated into functional neurons and respond to the inflammatory factor GrB similar to primary cultured human fetal neural progenitor cells. The system can be used for studying the pathophysiology of neuroinflammatory disorders using autologous inflammatory and brain cells on a personalized basis. The therapeutic relevance of using iNS for transplantation in neurodegenerative diseases needs further study.

## Experimental Procedures and Methods

### Ethics Statement

IRB exemptions were obtained for using anonymized human samples for this research from the Office of Human Subjects Research at the National Institutes of Health (NIH). Accordingly, human fetal brain specimens of 7-8 weeks gestation were obtained from Birth Defects Research Laboratory, University of Washington (Exempt No.:5831). Leukapheresis samples from deidentified adult healthy donors were obtained from Transfusion Medicine Blood Bank of the National Institutes of Health (Exempt No.: 11749). 

### Materials

Culture media and components were purchased from Invitrogen (Carlsbad, CA) and growth factors and cytokines were purchased from PeproTech (Rocky Hill, NJ) if not specified. 

### Purification of CD34+ cells

Blood from adult healthy donors was collected at Transfusion Medicine Blood Bank of the National Institutes of Health. Signed informed consent was obtained in accordance with the NIH Institutional Review Board.

CD34^+^ cells were purified either by positive selection from cells obtained via leukapheresis using a CD34 MultiSort Kit (Miltenyi Biotec, Bergisch Gladbach, Germany) according to manufacturer’s instructions or by negative selection on lineage depleted whole blood using a cocktail of monoclonal antibodies (mAbs) directed against CD2, CD3, CD14, CD16, CD19, CD24, CD36, CD56, CD66b (Stemcell Technologies, Vancouver, Canada) according to manufacturer’s instructions. The CD34+cells isolated by negative selection were washed in RPMI 1640 without serum (R0) medium and surface stained with a mixture of pre-titered amounts of directly conjugated mAbs to CD3-FITC, CD19-PerCP-Cy5.5 or CD19-brilliant violet 421, CD4-PE, CD8-FITC, CD34-PECy7, CD45-V500 (BD Biosciences, San Jose, California) in a total volume of 100 µl of R0 medium. Cells were stained for 20 min at 4°C. Cells were then washed, resuspended in 250 µl of PBS, and passed through a 40 micron strainer to assure single cell suspension. Flow cytometric analysis was done directly following this treatment and between 82% and 97% of CD34 purity was detected. Moreover, specific marker to detect mesenchymal cells, as CD29 and CD271, were used to stain the isolated CD34+ to further asses their purity.

### CD34+ cell culture and transfection

Cord blood CD34+ cells (purchased from Allcells, Emeryville, California) or adult CD34+ cells, isolated as previously described, were resuspended in StemSpan SFEM medium(Stemcell technologies) containing human thrombopoietin (TPO, 100 ng/ml), fms-like tyrosine kinase 3 (Flt-3) ligand (100 ng/ml) and stem cell factor (SCF, 100 ng/ml), interleukin-6 (IL-6, 20 ng/ml) and interleukin-7 (IL-7, 20 ng/ml) and cultured in a 6 well plate (3x10^5^/well in 2 ml of medium per well) at 37 °C in 5% CO_2_ incubator. Cells were replated daily if any adherent cells were observed to exclude non-hematopoietic population. Media were half changed every day by carefully removing the top 1 ml of the media as the cells were all floating in the bottom of the well. After 3 days, the cells were collected and centrifuged at 170 g for 10 min. 1x10^5^ cells/well in 200 ul were seeded in a 24 well plate. The cells were infected with 500 ul of CytoTune Sendai viral particles (Invitrogen) in pre-warmed media containing transcription factor constructs of hOct3/4, hSox2, hKlf4 and hc-Myc with an MOI of 3 for CD34+ cells from cord blood and MOI of 20 for adult peripheral blood CD34+ cells. Half media exchanges were performed every other day. Five days after transfection, the adherent cells were transferred to 6 well plates in neural progenitor cell medium (Dulbecco's Modified Eagle Medium: Nutrient Mixture F-12, DMEM/F12, containing 1xN2 supplement, 0.1% (w/v) bovine serum albumin (Sigma), 1% (v/v) antibiotics, 20 ng/ml of basic fibroblast growth factor (bFGF), and 20 ng/ml of Epidermal growth factor (EGF). When reaching 60% confluent, usually within a week, the cells were passaged at a ratio of 1: 3 by brief treatment with ethylenediaminetetraacetic acid (EDTA, 0.5 uM, Invitrogen) followed by mechanical dissociation. The cells were then transferred to new 6 well plates in neural stem cell medium (StemPro® NSC SFM - Serum-Free Human Neural Stem Cell Culture Medium, Invitrogen) for expansion. 

### T cell activation and co-culture with neural stem cells

T-cells were isolated via leukapheresis using a pan T cell isolation kit with MACS beads (Miltenyi Biotec) according to the manufacturer’s instructions and then incubated at 37°C in Iscove's modified Dulbecco's medium with 5% pooled human serum. The cells were activated by adding CD3/CD28 Dynabeads (Invitrogen) with a bead-to-cell ratio of 1:2 and incubated for 72 hours. T cells were then pelleted and collected by centrifugation at 210 g for 10 min. T cell- neural stem cell coculture was achieved by adding 2:1 T cells onto neural stem cells in 24 well plates. 

### Cell proliferation assay

The effect of GrB on the viability of neural stem cells was determined by a CellQuanti-blue Cell Viability Assay kit (BioAssay Systems, Hayward, CA). Briefly, cells were cultured in neural stem cell maintaining medium in 96-well plates and used for experiments at ^~^60% confluence. After adding recombinant GrB (1–10 nm; EMD Chemicals, Gibbstown, NJ), the cells were cultured for 24 hours. CellQuanti-blue solution (10 μl/well) was added and the cells were incubated for 30 minutes. The fluorescence was quantified at an excitation wavelength of 530 nm and emission wavelength of 590 nm using a fluorescence plate reader. Cell proliferation was measured by EdU incorporation assay. After 20 hours of GrB treatment, cells were treated with 10 uM of EdU (Invitrogen) for another 4 hours. The cells were then fixed with 4% (w/v) paraformaldehyde (Sigma) and EdU labeling was detected using Click-iT® EdU Imaging Kits (Invitrogen) according to the manufacturer’s instructions. DAPI was used for nuclear staining. Images of nine predetermined fields from each treatment group were taken and cell proliferation was determined by calculating the ratio of EdU positive cells and total DAPI positive cells. 

### Neural cell differentiation

For neuronal differentiation, cells were subcultured in 24 well plates at 1x10^5^/ml with neuronal differentiation medium [62] (DMEM/F12 containing 1xN2 supplement , 1xB27 supplement, 300 ng/ml cAMP (Sigma) and 0.2 mM vitamin C (Sigma), 10 ng/ml BDNF and 10 ng/ml GDNF ) for at least 14 days. The medium was changed twice a week. All growth factors were freshly added before each experiment. The growth factor containing medium was used within 1 week when stored at 4°C. For astroglial proliferation, the cells were seeded in 24 well plates at 1x10^5^/ml in neural stem cell medium. When cell attached after 24 hours of seeding, the media were replaced with astroglial differentiation medium containing DMEM/F12 with 10% fetal bovine serum (FBS) for 2 weeks. For oligodendrocytes differentiation, the cells were cultured on poly-L-Ornithine (Sigma) coated 24 well plates with oligodendrocyte differentiation medium consisting of DMEM/F12 containing N2 supplement (Invitrogen), 10 ng/ml of PDGF-AA, 2 ng/ml of NT-3, 2 ng/ml of Shh, and 3 nM of T3 (Sigma) for at least 14 days . The medium was then switched to DMEM/F12 with 1xN2 supplement and 3 nM of T3 and cultured for an additional week for MBP production. Neural differentiation were determined by immunostaining the cells for neuronal markers βIII-tubulin, NeuN, tyrosine hydroxylase, astroglial marker GFAP and oligodendrocyte markers O4 and MBP.

### Single cell colony formation and neural differentiation assays

Single cell colony formation and neural differentiation assays were used to confirm the stemness and tri-potency of induced neural stem cells. Published neural colony-forming cell assay protocols used for mouse neural stem cells [63,64] were adapted for the human neural stem cells. Briefly, monolayers of induced neural stem cells were dissociated into single cells by treatment with accutase and seeded at 1000 cells per milliliter (2000 cells per well) in a 6-well plate in collagen semisolid medium consisting of neural stem cell medium and collagen (Stem cell Technologies). After 14 days, each formed neural sphere (> 50 µm in diameter) was picked and dissociated into single cells for culture of secondary colonies in the collagen semisolid culture. After 14 days, formed secondary neurospheres (> 50 µm in diameter) were counted and picked individually and dissociated. Cells from each sphere were seeded into two wells of 48-well plate coated with poly-D-lysine with astroglial differentiation medium or oligodendrocyte differentiation medium for 7 days. Neural cell differentiation was analyzed by immunostaining. 

### Cultures of other neural stem cells

 To generate iPSC from adult CD34+ cells, we followed the published protocol[48] with modifications. Five days after Sendai virus infection, CD34+ cells were transferred to Cellstart coated 24 well plates in STEMPRO Human Embryonic Stem Cell Culture Medium (Invitrogen). The iPSC colonies were picked three weeks later and expended in the same culture condition. IPSC characterization was performed using hES/iPS cell characterization kit (Applied Stemcell, Sunnyvale, CA). To induce neural stem cell differentiation, iPSC were dissociated using EDTA and seeded on a 6 well plate without coating in neural stem cell medium. The attached neural stem cells were expended and used before passage 3. 

Human neural progenitor cells (NPC) were cultured following previous published paper [6]. NPC were used at passage 3.

### Immunocytochemistry

Cells were fixed in 4% paraformaldehyde for 10 min at room temperature, followed by 3 times of washing with PBS. After incubation in 0.1% (v/v) Triton X-100 in PBS (PBS-T) for 10 min at room temperature, the cells were incubated with blocking buffer (PBS-T containing 4% (v/v) goat serum and 1% (v/v) glycerol (Sigma)) at room temperature for 20 min. Cells were immunostained with mouse monoclonal anti-Nestin (Clone 10C2, 1:1000; Millipore Billerica, MA), mouse monoclonal anti-β-III-tubulin (1:1000; Promega, Madison, WI), rabbit anti-PAX6 (1:200, Abcam), rabbit anti-vGLUT1 (1:200, Abcam), rabbit anti-vGAT (1:200, Abcam) rabbit anti-TH (1: 1000, Novus) rabbit anti-GFAP (1:1000, Sigma), mouse monoclonal anti-oligodendrocyte marker O4 (1:200, R&D system, Minneapolis, MN), chicken anti-MBP antibody (1:200, Millipore) and ready to use rabbit anti-SOX-2 and anti-OCT4 antibodies from hES/iPS cell characterization kit (Applied Stemcell), followed by corresponding secondary antibodies (anti-mouse Alexa Fluor 488, 1:400; anti-rabbit Alexa Fluor 594, 1:400; anti-chicken Alexa Fluor 594, 1:400; Invitrogen) and DAPI nuclear staining. Images were acquired on a Zeiss LSM 510 META multiphoton confocal system (Carl Zeiss) or EVOS fluorescence microscope (AMG, Bothell, WA). 

### Microarray analysis

RNA extraction, isolation, quality control (QC), quantitation, cRNA synthesis and labeling were performed on a sample-by-sample basis according to manufacturer’s guidelines for use with the Human GeneChip 2.0 ST Array (Affymetrix, Inc). Cell lysis, on-column DNA digestion and RNA purification were performed using Total RNA purification kit (Norgen Biotek, Canada) following the kit instruction. Bioanalyzer nanochip (Agilent, Inc) and NanoDrop (Agilent, Inc) were used to validate and quantitate the RNA. For cRNA synthesis and labeling, 200 ng of total RNA was used per sample in conjunction with the Ambion WT Expression Kit (cat#4411973) and Affymetrix WT Terminal labeling Kit (cat#901525). Labeled cRNA were hybridized to the Affymetrix Human GeneChip 2.0 ST Array (Affymetrix, Inc) over two separate runs in blinded interleaved fashion. The Affymetrix scanner 3000 was used in conjunction with Affymetrix GeneChip Operation Software to generate probe-level data for each hybridized cRNA. Probe-level data summarization and normalization was accomplished using the Affymetrix Expression Console with the "RMA Sketch" option selected (Affymetrix, Inc).  Resulting gene fragment data was first baseline subtracted to correct for run-to-run differences using class means.  After, data quality was inspected and assured via sample-level Tukey box plot, covariance-based PCA scatter plot and correlation-based clustering using the “box.plot”, “princomp”, “cor”, and “heatmap.2” functions supported in R (http://cran.r-project.org/).  System noise for the data was defined to equal the mean expression value across all experiment samples at which the observed Coefficient of Variation (CV) of expression by “lowess” fit grossly deviated from linearity (6.25).  Gene fragments not having at least one expression value greater than system noise were discarded.  Gene fragments not discarded were floored to system noise if less than system noise and annotated using IPA (www.Ingenuity.com).  Gene fragments with known gene annotation were next subset and intersected with pluripotency and neuronal progenitor markers (NCBI PMID: 23117585) which were listed as Table S1. Sample to sample relationships based on the intersection were interrogated by covariance-based PCA scatter plot and correlation-based clustering in R.  Significant differences in gene expression for intersected neuronal progenitor markers were subsequently identified in R via Welch-modified t-test under Benjamini-Hochberg multiple comparison correction condition using the t.test and multtest functions respectively.  Such that, a neuronal progenitor marker having both a corrected p-value < 0.05 and an absolute difference of means between iNS and NPC were construed to have expression significantly different between iNS and NPC for the marker. Corresponding data files can be found via this url: http://www.ncbi.nlm.nih.gov/geo/query/acc.cgi?acc=GSE44532

### Electrophysiology

Whole –cell patch clamp recordings were performed at room temperature from cells after 14-16 days of neuronal differentiation induction. The extracellular recording bath contained (in mM) 145 NaCl, 3 KCl, 10 4-(2-hydroxyethyl)-1-piperazineethanesulfonic acid (HEPES), 2 CaCl_2_ , 2 MgCl_2_ and  8 glucose (pH 7.2, 300 mOsm). The recording electrodes (4-8 MΩ resistance) were filled with an internal solution containing (in mM): 125 K-gluconate, 20 KCl, 10 HEPES, 4 NaCl, 0.5 ethylene glycol tetraacetic acid (EGTA), 4 Mg ATP, 0.3 GTP, 10 phosphocretine (pH 7.2, 290 mOsm). Electrophysiological recordings were obtained using a multiclamp 700B amplifier and PClamp 10 (Molecular Devices, Sunnyvale, CA). Data were analyzed using IGOR Pro (WaveMetrics, Lake Oswego, OR).

### Electron microscopy sample preparation

The neural stem cells and activated T cells were co-cultured in a gridded glass bottom petri dish (MatTek Corp). The culture medium was replaced with three PBS exchanges then fixed in 2% gluteraldehyde in 0.1 M sodium cacodylate buffer pH 7.0 for 1 hour at room temperature. The sample was washed with sodium cacodylate buffer three times for 10 minutes each. The cells were post-fixed with 1% osmium tetroxide in sodium cacodylate for 1 hour at room temperature and washed with sodium cacodylate twice and 0.1 M sodium acetate pH 4.2 buffer once for 10 minutes each. The cells were stained *en bloc* with 0.5% uranyl acetate in sodium acetate buffer for 1 hour in room temperature followed by three washes in sodium acetate for 10 minutes each. The sample was dehydrated in two 10 minutes incubations with 35%, 50%, 70%, and 95% ethanol/deionized water followed by three 10 minutes incubations with 100% Ethanol in room temperature. The ethanol was exchanged with 100% resin using PolyBed 812 Luft formulations Embedding Kit/DMP-30 (PolySciences) three times for 10 minutes each. The sample was left in the chemical hood on a gyrator overnight. The next day, the resin was replaced with freshly made resin and placed in 55°C oven for 48 hours to induce polymerization. Hydrofluoric acid 48 wt% was used to remove the glass bottom. The resin block was removed from the petri dish using a Dremel (Bosch) and the debris was removed using a sonicator for 6 minutes in deionized water. Finally, the embedded resin block was mounted on a specimen stub using colloidal silver paint (Electron Microscopy Sciences) and sputter coated with gold.

### Data collection with Ion Abrasion Scanning Electron Microscopy (IA-SEM)

A Zeiss NVision 40 Crossbeam Microscope (Carl Zeiss NTS) equipped with Atlas3D (Fibics Inc., Ottawa) was used for data collection. The resin block was imaged with an electron beam at 15 kV to locate neural stem cell and T cell conjugates. The sample stub was tilted to 54° and a 1 um of carbon layer was deposited on the region of interest using a gas injection system with a focused ion beam of 30 kV and 300 pA. A trapezoidal trench was created using a13nA ion beam and the cliff face was polished with a 300pA ion beam in front of the region interest. A 300 pA focused ion beam iteratively removed 16 nm slices from the cross section while an electron beam scanned the newly revealed surface at 4 nm per pixel. The images were recorded using an energy selective back scattered electron (ESB) detector at 1.5 kV with an aperture size of 60 um. The result is a stack of 545 two-dimensional scanning electron microscopy images capturing a region of interest of 17.57 um x 6.30 um x 8.72 um.

### Processing of Electron Microscopy Images

The individual 2D tiff images were merged into a single mrc stack, then cropped and aligned using customized scripts based on the image processing program IMOD (UC Boulder). Features of interest were automatically selected with 3DSlicer using a threshold tool that highlighted features within a specified range of pixel intensity. The automatic segmentation was polished manually using Avizo Fire (Visualization Sciences Group). The 2D segmentations were converted into polygons to produce 3D visualizations using Autodesk 3D Studio Max software. 

### Statistics

For experiments that required immunostaining, the total number of cells and immunolabeled cells were counted in nine predetermined fields per coverslip or well using a fluorescence microscope with a 20× objective. Three coverslips/wells were counted in each group. At least three independent experiments were performed. Mean and standard error (SE) were calculated for each treatment group. Statistical analysis was performed using Prism, version 3.0. Differences were tested using one-way ANOVA followed by Bonferroni's test for multiple groups and Students T test for two groups. Two-tailed values of *p* < 0.05 were considered significant. 

## Supporting Information

Figure S1 **Sample-to-sample relationships based on correlation-based clustering analysis using 65 neuronal progenitor markers (NCBI PMID: 23117585).** Markers represented have a Benjamini-Hochberg corrected Welch-modified t-test p-value < 0.05 and an absolute difference of means when iNS and NPC samples are compared.  Both the clustering analysis and significance testing was performed in R (http://cran.r-project.org/) using the heatmap.2, t.test and multtest functions respectively.  Marker expression depicted is of type RMA (log, base=2).
(PDF)

Figure S2 **Confirmation of neural cell markers using Western-blot assay.** PAX6 protein production in iNS was studied using Western-blot. Neural stem cells derived from a characterized iPS cell line was used as control. Similar pattern of PAX6 expression was observed. MBP production in oligodendrocytes which were differentiated from iNS was also confirmed using Western-blot assay.
(PDF)

Figure S3 **Neural stem cell signature analysis.** GeneSet Enrichment Analysis (GSEA) was used to test whether neural stem cell gene signature is significantly enriched for genes differentially expressed between iNS and parental CD34 cells. Description of GSEA can be found at http://www.broadinstitute.org/gsea/. All genes on the microarray are ranked by fold change (iNS/CD34), and the GSEA algorithm overlay established gene sets signature over the microarray ranked list. For each gene on the gene set, vertical bars along the x-axis of the GSEA plot represent the position of genes within the ranked list. Based on the number of genes from the gene set that hit the highly ranked gene on the microarray list, an Enrichment Score (ES) and p-value is computed (Green plot). As there is no pre-defined neural stem cell gene signature set in GSEA database so we went through Medline GEO data sets and generated a couple of gene signatures from GSE38045 (http://www.ncbi.nlm.nih.gov/geo/query/acc.cgi?acc=GSE38045) and GSE37832 (http://www.ncbi.nlm.nih.gov/geo/query/acc.cgi?acc=GSE37832). INS correlate with direct-generated neural stem cells (iNSC) from human fibroblasts in GSE38045 and mouse adult neural stem cells from the subventricular zone of 3rd ventricle in GSE37832, showing high enrichment score (ES) and statistical significance. For neural stem cells from human fibroblasts, the ES for the up-regulated genes was 0.72 (p<0.0001) and for the neural stem cells from the subventricular zone of 3rd ventricle the ES for the up-regulated genes was 0.67 (p<0.0001). These results indicate that iNS generated from CD34+ cells shared similarity with neural stem cells generated from fibroblasts and adult neural stem cells from subventricular zone of 3rd ventricle.
(PDF)

Figure S4 **Live imaging of neural stem cell membrane markers.** When reaching 60% confluence, iNS cells at passage 42 were incubated with mouse monoclonal antibodies against neural stem cell surface markers CD15 (1: 100, Abcam) or CD24 (1:100, Abcam) and rabbit polyclonal antibody against astroglial cell surface marker CD44 (1:100, Abcam) for 1 hour at room temperature. After washing with fresh media, the cells were incubated with corresponding secondary antibodies (anti-mouse or anti-rabbit Alexa Fluor 488, 1:400) for 1 hour. After washing with fresh media, the cells were live imaged under a fluorescence microscope (AMG). The representative images were presented to show that most of the cells were still positive for neural stem cell surface marker CD15 (A) and CD24 (B) but not for astroglial marker CD44 (C).
(PDF)

Figure S5 **Colony formation and neural cell differentiation from single neural stem cells.** Single cell derived colony formation was achieved by seeding low density of isolated cell solution (1000 cells/well) in collagen semisolid medium. Additional 1 ml of neural stem cell medium was added each week to counter evaporation. After 14 days, each primary neural stem cell colony (> 50 µm in diameter, 1st) was collected and dissociated into single cells for culture of the secondary neurospheres (2nd, A). Cells from an individually collected secondary neurosphere were dissociated and seeded into two wells of a 48-well-plate. One well of cells was cultured in astroglial differentiation medium and the other was cultured in oligodendrocyte differentiation medium. After 4-7 days, differentiated cells were immunostained for βIII-tubulin, GFAP and O4. Representative images showed that βIII-tubulin, GFAP and O4 positive cells were derived from one colony (B).
(PDF)

Figure S6 **Immunophenotype analysis performed on the enriched isolated CD34+ cells.** . Flow cytometric analysis enriched CD34+ cells was done as described in Methods. CD34+ cells are represented in the first plot. The same CD34+ cells were stained for specific markers to detect mesenchymal cells. CD29 and CD271 was negative on the isolated CD34+ cells. BV421, brilliant violet; PE, phycoerythrin; PerCP-Cy5.5, peridinin-chlorophyll protein complex.
(PDF)

Table S1 **List of genes included in pluripotent gene set and neural progenitor gene set.**
(XLSX)
