# Correction: Derivation of Neural Stem Cells from Human Adult Peripheral CD34+ Cells for an Autologous Model of Neuroinflammation

**DOI:** 10.1371/journal.pone.0091277

**Published:** 2014-03-21

**Authors:** The PLOS ONE Staff

The 13th author's name is spelled incorrectly. The correct name is: Dax A. Hoffman.
