# Supplementary figures and images for: Derivation of Neural Stem Cells from Human Adult Peripheral CD34+ Cells for an Autologous Model of Neuroinflammation
Source: PLoS One. 2013 Nov 26;8(11):e81720. doi: 10.1371/journal.pone.0081720 (PMC3841177; doi:10.1371/journal.pone.0081720)

Figure S1

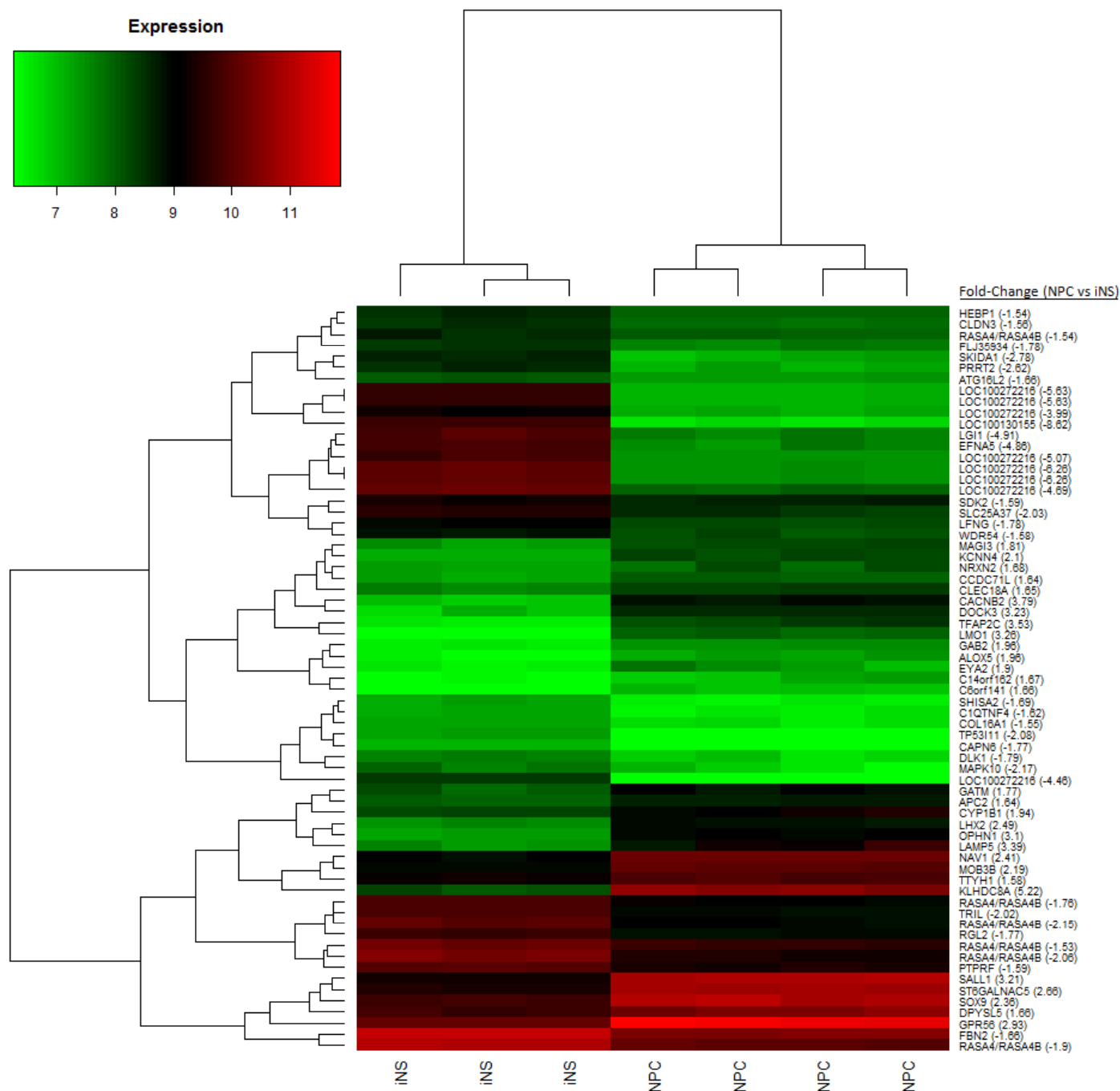

Supplement: Figure S1 — Sample-to-sample relationships based on correlation-based clustering analysis using 65 neuronal progenitor markers (NCBI PMID: 23117585). Markers represented have a Benjamini-Hochberg corrected Welch-modified t-test p-value < 0.05 and an absolute difference of means when iNS and NPC samples are compared. Both the clustering analysis and significance testing was performed in R (http://cran.r-project.org/) using the heatmap.2, t.test and multtest functions respectively. Marker expression depicted is of type RMA (log, base=2). (PDF) [file pone.0081720.s001.pdf]

Figure S2

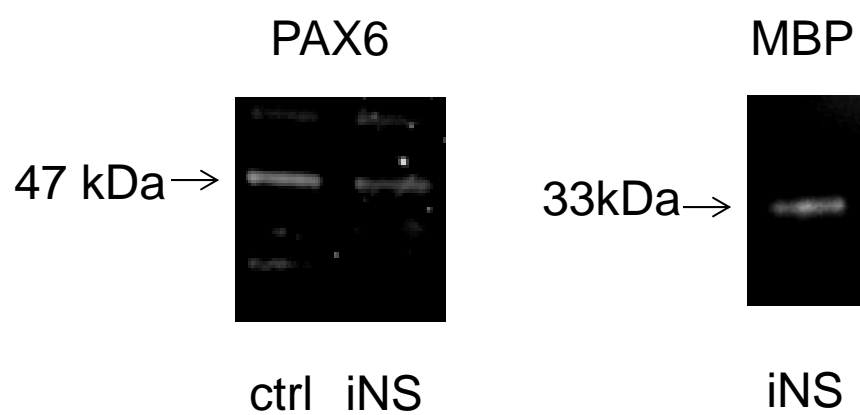

Supplement: Figure S2 — Confirmation of neural cell markers using Western-blot assay. PAX6 protein production in iNS was studied using Western-blot. Neural stem cells derived from a characterized iPS cell line was used as control. Similar pattern of PAX6 expression was observed. MBP production in oligodendrocytes which were differentiated from iNS was also confirmed using Western-blot assay. (PDF) [file pone.0081720.s002.pdf]

Figure S4

P42

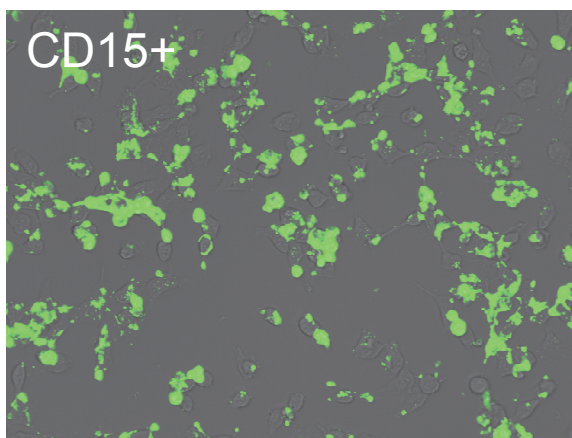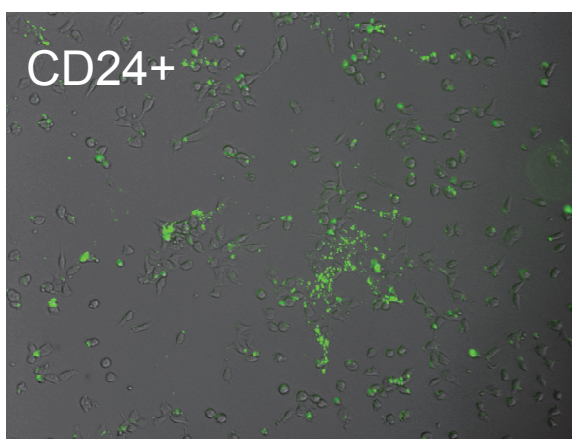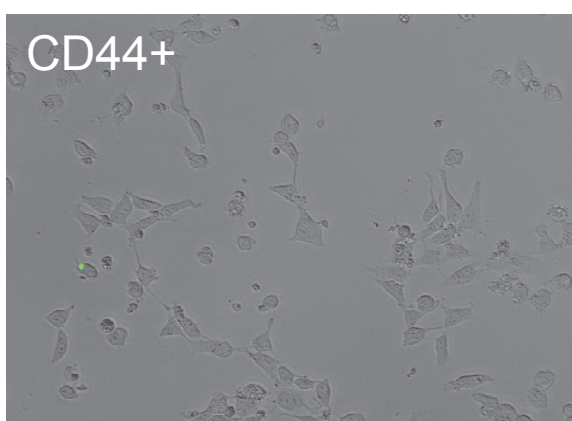

Supplement: Figure S4 — Live imaging of neural stem cell membrane markers. When reaching 60% confluence, iNS cells at passage 42 were incubated with mouse monoclonal antibodies against neural stem cell surface markers CD15 (1: 100, Abcam) or CD24 (1:100, Abcam) and rabbit polyclonal antibody against astroglial cell surface marker CD44 (1:100, Abcam) for 1 hour at room temperature. After washing with fresh media, the cells were incubated with corresponding secondary antibodies (anti-mouse or anti-rabbit Alexa Fluor 488, 1:400) for 1 hour. After washing with fresh media, the cells were live imaged under a fluorescence microscope (AMG). The representative images were presented to show that most of the cells were still positive for neural stem cell surface marker CD15 (A) and CD24 (B) but not for astroglial marker CD44 (C). (PDF) [file pone.0081720.s004.pdf]

Figure S5

**A**

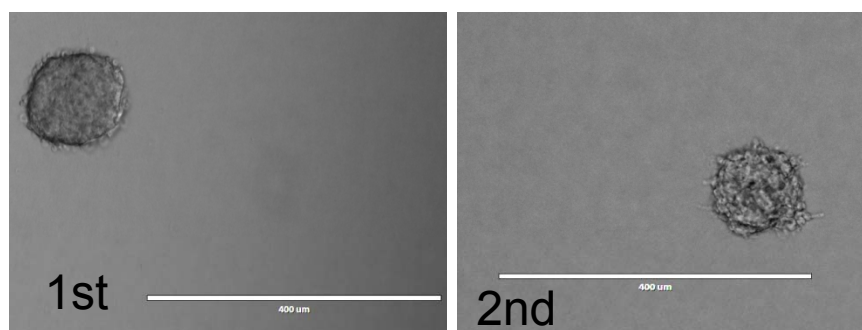

**B**

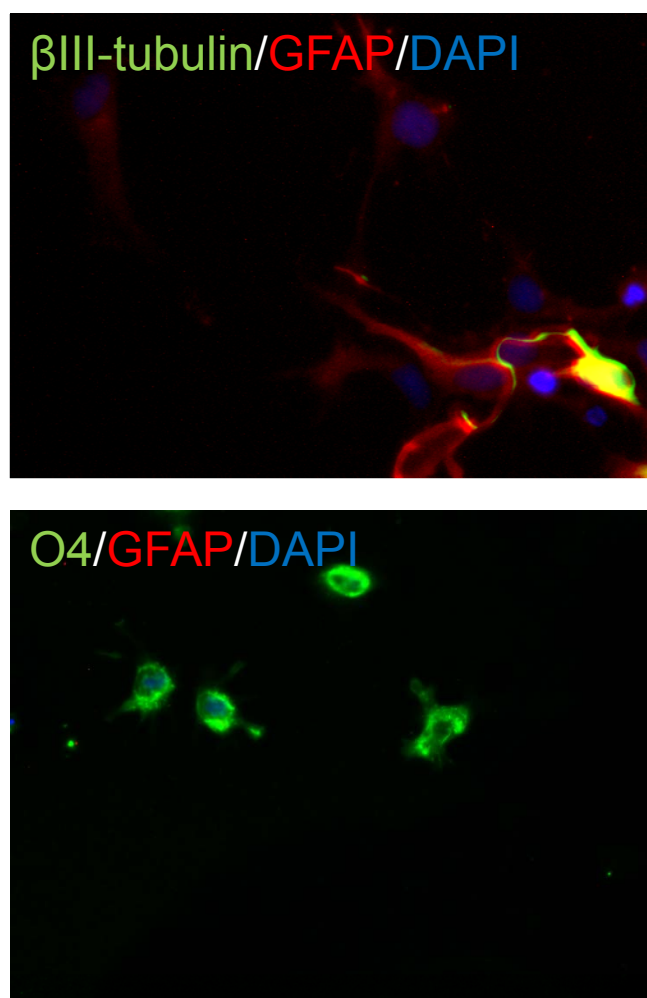

Supplement: Figure S5 — Colony formation and neural cell differentiation from single neural stem cells. Single cell derived colony formation was achieved by seeding low density of isolated cell solution (1000 cells/well) in collagen semisolid medium. Additional 1 ml of neural stem cell medium was added each week to counter evaporation. After 14 days, each primary neural stem cell colony (> 50 µm in diameter, 1st) was collected and dissociated into single cells for culture of the secondary neurospheres (2nd, A). Cells from an individually collected secondary neurosphere were dissociated and seeded into two wells of a 48-well-plate. One well of cells was cultured in astroglial differentiation medium and the other was cultured in oligodendrocyte differentiation medium. After 4-7 days, differentiated cells were immunostained for βIII-tubulin, GFAP and O4. Representative images showed that βIII-tubulin, GFAP and O4 positive cells were derived from one colony (B). (PDF) [file pone.0081720.s005.pdf]

Figure S6

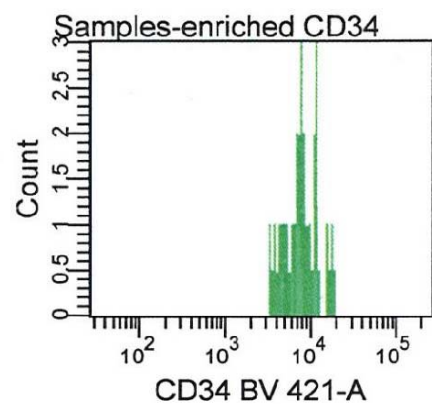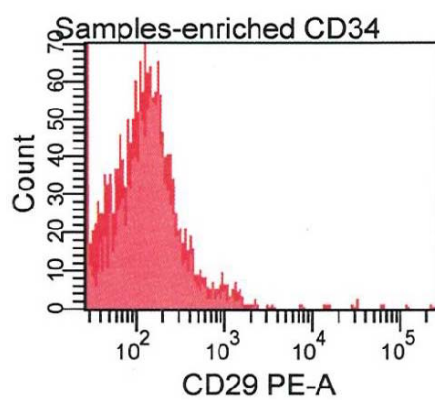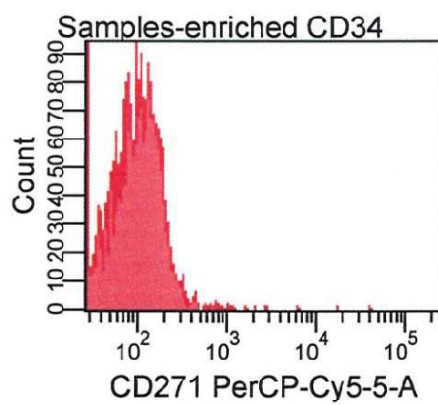

Supplement: Figure S6 — Immunophenotype analysis performed on the enriched isolated CD34+ cells. . Flow cytometric analysis enriched CD34+ cells was done as described in Methods. CD34+ cells are represented in the first plot. The same CD34+ cells were stained for specific markers to detect mesenchymal cells. CD29 and CD271 was negative on the isolated CD34+ cells. BV421, brilliant violet; PE, phycoerythrin; PerCP-Cy5.5, peridinin-chlorophyll protein complex. (PDF) [file pone.0081720.s006.pdf]
