# Supplementary material for: Derivation of Neural Stem Cells from Human Adult Peripheral CD34+ Cells for an Autologous Model of Neuroinflammation
Source: PLoS One. 2013 Nov 26;8(11):e81720. doi: 10.1371/journal.pone.0081720 (PMC3841177; doi:10.1371/journal.pone.0081720)
Supplement: Figure S3 — Neural stem cell signature analysis. GeneSet Enrichment Analysis (GSEA) was used to test whether neural stem cell gene signature is significantly enriched for genes differentially expressed between iNS and parental CD34 cells. Description of GSEA can be found at http://www.broadinstitute.org/gsea/. All genes on the microarray are ranked by fold change (iNS/CD34), and the GSEA algorithm overlay established gene sets signature over the microarray ranked list. For each gene on the gene set, vertical bars along the x-axis of the GSEA plot represent the position of genes within the ranked list. Based on the number of genes from the gene set that hit the highly ranked gene on the microarray list, an Enrichment Score (ES) and p-value is computed (Green plot). As there is no pre-defined neural stem cell gene signature set in GSEA database so we went through Medline GEO data sets and generated a couple of gene signatures from GSE38045 (http://www.ncbi.nlm.nih.gov/geo/query/acc.cgi?acc=GSE38045) and GSE37832 (http://www.ncbi.nlm.nih.gov/geo/query/acc.cgi?acc=GSE37832). INS correlate with direct-generated neural stem cells (iNSC) from human fibroblasts in GSE38045 and mouse adult neural stem cells from the subventricular zone of 3rd ventricle in GSE37832, showing high enrichment score (ES) and statistical significance. For neural stem cells from human fibroblasts, the ES for the up-regulated genes was 0.72 (p<0.0001) and for the neural stem cells from the subventricular zone of 3rd ventricle the ES for the up-regulated genes was 0.67 (p<0.0001). These results indicate that iNS generated from CD34+ cells shared similarity with neural stem cells generated from fibroblasts and adult neural stem cells from subventricular zone of 3rd ventricle. (PDF) [file pone.0081720.s003.pdf]

Figure S3

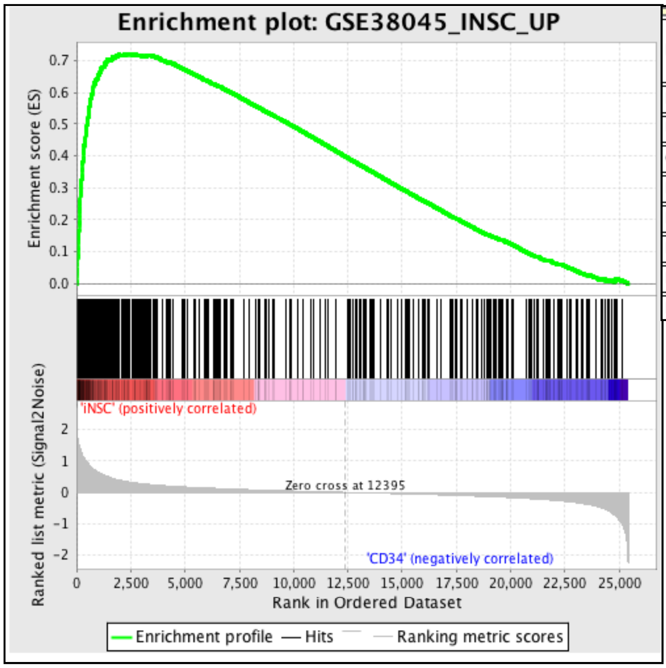

Dataset	DWang_GSEA022413_GSEA022413.iNSC_vs_CD34.cls #iNSC_versus_CD34.iNSC_vs_CD34.cls #iNSC_versus_CD34_repos
Phenotype	iNSC_vs_CD34.cls#iNSC_versus_CD34_repos
Upregulated in class	iNSC
GeneSet	GSE38045_INSC_UP
Enrichment Score (ES)	0.7212686
Normalized Enrichment Score (NES)	1.0734679
Nominal p-value	0.0
FDR q-value	0.07713499
FWER p-Value	0.0

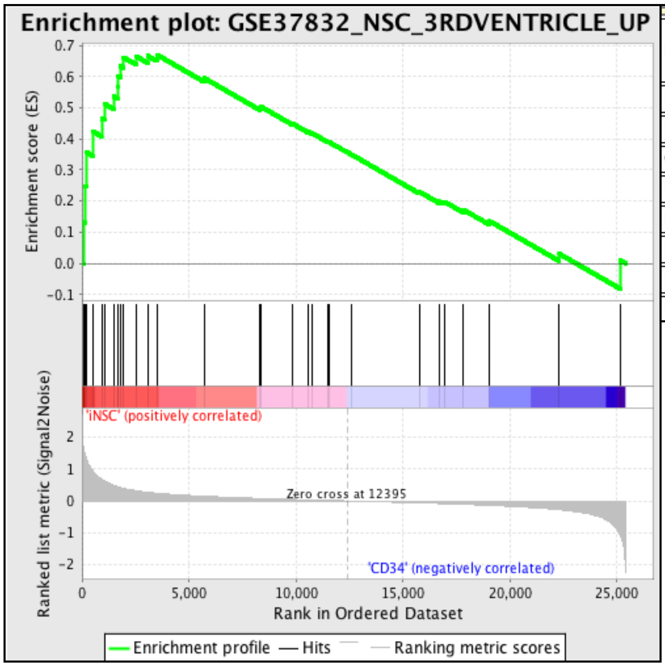

Dataset	DWang_GSEA022413_GSEA022413.iNSC_vs_CD34.cls #iNSC_versus_CD34.iNSC_vs_CD34.cls #iNSC_versus_CD34_repos
Phenotype	iNSC_vs_CD34.cls#iNSC_versus_CD34_repos
Upregulated in class	iNSC
GeneSet	GSE38045_INSC_UP
Enrichment Score (ES)	0.7212686
Normalized Enrichment Score (NES)	1.0734679
Nominal p-value	0.0
FDR q-value	0.07713499
FWER p-Value	0.0
